# A generative co-design framework for healthcare innovation: development and application of an end-user engagement framework

**DOI:** 10.1186/s40900-021-00252-7

**Published:** 2021-03-01

**Authors:** M. Bird, M. McGillion, E. M. Chambers, J. Dix, C. J. Fajardo, M. Gilmour, K. Levesque, A. Lim, S. Mierdel, C. Ouellette, A. N. Polanski, S. V. Reaume, C. Whitmore, N. Carter

**Affiliations:** 1 School of Nursing, McMaster University, 1280 Main St. W., Hamilton, ON L8S 4L8 Canada; 2 DigiComp Kids, Hamilton, Canada; 3 VON Canada, 2315 St. Laurent Blvd, Suite 100, Ottawa, ON K1G 4J8 Canada; 4 Ontario Health (OTN), 438 University Avenue, Suite 200, Toronto, ON M5G 2K8 Canada; 5 D’Youville College, Patricia H. Garman School of Nursing, Buffalo, USA; 6 Department of Pediatrics, McMaster University, 1280 Main St. W., Hamilton, ON L8S 4L8 Canada; 7 McMaster University, 1280 Main St. W., Hamilton, ON L8S 4L8 Canada; 8 School of Public Health and Health Systems, University of Waterloo, 200 University Ave West, Waterloo, ON N2L 3G1 Canada

**Keywords:** End-user engagement, Healthcare innovation, Co-design, Patient and public involvement

## Abstract

**Plain English summary:**

**Background**

Continual improvements to health systems, products, and services are necessary for improvements in health. However, many of these improvements are not incorporated into everyday practice. When designing new health systems, products, and services, involving members of the healthcare community and the public with personal healthcare experience can help to make sure that improvements will be useful and relevant to others like them.

**Methods**

Together with healthcare workers and family members with healthcare experience, we developed and applied a step-by-step guide to involving those with personal experience in the design of health system improvements.

**Results**

Our guide has three phases— ‘Pre-Design’, ‘Co-Design’, and ‘Post-Design’. This paper describes each of these phases and illustrates how we applied them to our own project, which is to use virtual healthcare methods to improve care for children with chronic healthcare conditions and their families. In our own work, we found that healthcare workers and family members with personal healthcare experiences were able to use their knowledge and creativity to help us imagine how to improve care for children with chronic healthcare conditions and their families. We have created action items from these family member- and healthcare worker-identified needs, which we will use to shape our virtual healthcare system.

**Conclusions**

This paper may be useful for those seeking to involve members of the healthcare community and the public in the creation of better healthcare systems, products, and services.

**Abstract:**

**Background**

Challenges with the adoption, scale, and spread of health innovations represent significant gaps in the evidence-to-practice cycle. In the health innovation design process, a lack of attention paid to the needs of end-users, and subsequent tailoring of innovations to meet these needs, is a possible reason for this deficit. In the creative field of health innovation, which includes the design of healthcare products, systems (governance and organization mechanisms), and services (delivery mechanisms), a framework for both soliciting the needs of end-users and translating these needs into the design of health innovations is needed.

**Methods**

To address this gap, our team developed and applied a seven-step methodological framework, called A Generative Co-Design Framework for Healthcare Innovation. This framework was developed by an interdisciplinary team that included patient partners.

**Results**

This manuscript contributes a framework and applied exemplar for those seeking to engage end-users in the creative process of healthcare innovation. Through the stages of ‘Pre-Design’, ‘Co-Design’, and ‘Post-Design’, we were able to harness the creative insights of end-users, drawing on their experiences to shape a future state of care. Using an expository example of our own work, the DigiComp Kids project, we illustrate the application of each stage of the Framework.

**Conclusions**

A Generative Co-Design Framework for Healthcare Innovation provides healthcare innovators, applied health science researchers, clinicians, and quality improvement specialists with a guide to eliciting and incorporating the viewpoints of end-users while distilling practical considerations for healthcare innovation and design.

**Supplementary Information:**

The online version contains supplementary material available at 10.1186/s40900-021-00252-7.

## Background

Internationally, efforts to cultivate the inclusion of patient partners in research for the purposes of creating meaningful change in patient-important research outcomes have been driven by funding bodies, patient communities, and government initiatives [1]. In support of these growing calls to action, the literature base detailing practical techniques for operationalizing patient partnership in health research is expanding (examples [2–4]). A 2019 systematic review by Greenhalgh and colleagues summarized over 65 frameworks designed to support, evaluate, or report on patient partnerships in health research [5]. From these diverse frameworks, a taxonomy of five framework categories emerged, including frameworks that were focused on: power dynamics, priority setting, study processes, reporting, and supporting patient partnerships [5]. Concomitant with increasing efforts to include patient partners in the aforementioned research endeavours, the need to include end-users in health innovation efforts is becoming increasingly recognized. This includes engaging patient partners and other stakeholders invested in the outcomes of health innovations in creative health innovation efforts such as the design of healthcare products, systems, and services [6, 7]. When designing new health innovations, the value of creativity in the design process cannot be understated. Creativity is defined as the generation of new ideas, while design is a structured process, whereby creative ideas are transformed into specific products, systems, or services – serving as the link between creativity and innovation [8]. Environments which encourage and nurture highly creative ideas have been linked to more robust research and development efforts, which in turn drive more successful innovation outputs [8]. It is vital that innovation end-users including patients, caregivers, clinicians, and healthcare decision makers are included as partners within these creative innovation efforts, so that innovations may be influenced by end-users’ knowledge as ‘experts of their experiences’ [9], thus shaping health solutions to focus on end-user priorities, ultimately leading to better patient outcomes and greater uptake [10]. However, while a multitude of frameworks exist for gathering insight from end-users (e.g. the use of narrative interview techniques in Experience-Based Co-Design), none describe immersing end-users in highly creative environments and guiding them through a creative process of health innovation. Thus, end-user engagement frameworks specific to the field of health innovation and tailored toward creative innovation methodologies are largely absent in the literature. Building on the work of Greenhalgh et al. [5], we argue the need for a sixth category to be added to the taxonomy of patient partnership frameworks, focused on engaging end-users in the creative process of health innovation design. In what follows, we illustrate our search for, and subsequent construction of, an engagement framework for health innovation, using our work, The DigiComp Kids Project, as an expository example.

## Methods

### Our project: DigiComp Kids

As a research team committed to engaging patient partners and other end-users in our work, we sought a framework to guide us in involving end-users in our health innovation effort, the DigiComp Kids Project. The aim of the DigiComp Kids Project is to co-design, develop, and test a virtual care program that enables children with medical complexities to receive comprehensive, integrated healthcare at home. A key objective of this project is to leverage digital technology to connect family members to home-based and hospital-based clinicians who care for medically complex children. To do so, we partnered with Ontario Health, Ontario Telemedicine Network (OTN) business unit, with the goal of bringing families and healthcare teams together virtually, in order to deploy a seamless and integrated approach to care at home.

The existing model of care for children with medical complexities and their families at our centre involves family members as well as specialized home-based and hospital-based clinicians providing care independently, but without the means to effectively communicate and operate as one cohesive team. The hospital-based Complex Care Team provides specialized interdisciplinary care via clinic and inpatient services to children with medical complexities and their families, while home-based clinicians provide close monitoring and care at home. Telephone, fax, and email are all used to intermittently communicate among siloed teams when necessary. For example, a family member may telephone a Complex Care Team member to help troubleshoot a problem with a piece of equipment, or a clinician from the Complex Care Team may fax a communication about a medication change to be carried out by a home care clinician. One of the key goals for a new model of care was finding a way to streamline communication and care processes amongst team members.

### Our approach to co-design

To accomplish our project aims of integrating care and connecting care teams, we set out to co-design a virtual program that would allow for a seamless care experience for children with medical complexities and their families. Through our co-design process, we aimed to answer the question: What are the optimal processes, features, and workflows for a virtual care intervention to provide integrated home-based care for children with medical complexities? Table 1 (Additional file 1) contains the definitions used in our work for processes, features, and workflows.

From the beginning of the DigiComp Kids Project, our research team agreed that a core value to guide our work would be the engagement of our innovation end-users including patient and family partners, hospital-based and home-based clinicians, and system navigators in co-designing a new virtual care model. In addition, as we aimed to design a new model for healthcare service delivery, we were interested in methods that would accommodate for creative approaches to idea generation. A diverse body of literature has exposed the integral role that creativity has in innovation work, with some citing creativity as the most central component of the process of innovation [11, 12]. Within the creative design literature, a focus on generative techniques to solicit design requirements has emerged. Generative techniques aim to map participants’ latent needs and desires by allowing them to explore challenges and create alternative future scenarios by solving those challenges [9]. These techniques encourage participants to create an artefact, such as a story, about a future state in which present-day challenges are resolved. Through the creation of artefacts, participants are able to tap into their creative minds and express and harness their experiences to solve present day challenges. Generative techniques have been successfully used to gather patient insights in past examples of healthcare service design by allowing participants to imagine ‘alternative future scenarios’ or situations and contexts that are very different from their current reality [13]. By employing generative methodologies with individuals with lived experiences of healthcare challenges through the co-design process, healthcare innovations which are relevant, acceptable, and context-specific may be created.

In searching for an existing model that would guide us in our project, we reviewed the work of Greenhalgh [5] and others [14, 15] to select a suitable framework for including our end-users in creative design work for health innovation. Through our focused literature search, we found that our values of patient partner and stakeholder engagement aligned closely with values guiding three categories of engagement frameworks— Participatory Action Research, Community-Based Participatory Research, and Experience-Based Co-Design.

In Participatory Action Research, participants engage in critically reflective exercises to understand and change systems and situations in which they find themselves [16]. Researchers and participants in Participatory Action Research often seek to create more evenly distributed social justice via the actions taken through their work by using experience-based knowledge to change practice [17]. In Community-Based Participatory Research, the collaborative work of researchers and laypeople focuses on addressing community-identified needs [18]. An equitable relationship between laypeople and researchers ensues, with community members often helping to identify research priorities, as well as the research question and methods [19]. The ultimate goal of Community-Based Participatory Research is the generation of information that will benefit the community and support community capacity-building [19]. Finally, using Experience-Based Co-Design, patients and staff collaborate with researchers by participating in narrative interviews to detail their experiences with the current healthcare system and services, which are then used as a basis on which to improve future experiences for others [20].

While each of these frameworks provides valuable guidance for involving patient partners and stakeholders in the research process, none speak directly to the role of the creative process of end-users in health innovation. Operationally, we required guidance that specified how to channel deep engagement of end-users in co-design via creative methods, so as to reveal latent needs and generate alternative future scenarios. Our team thus developed and applied a new framework with these specific aims in mind, entitled— A Generative Framework for Healthcare Innovation. The three major stages of this framework (pre-design, co-design, and post-design) were conceptualized after our review of current frameworks revealed none that were suitable for our needs. Using these stages as a starting point, our team began the process of moving through these stages in turn, beginning with pre-design, and iteratively recording and refining the steps taken and operational decisions made in each phase. In this way, the phases of development and application of the framework reciprocally informed each other. In what follows, we describe the end-user engagement philosophy grounding our work, before presenting our seven-step methodological framework.

### End-user involvement in this study

End-user involvement is integral to the design, conduct, and analysis of the entire DigiComp Kids project, and is reported here according to the GRIPP2 Long Form Checklist [21] (Additional file 2). The central DigiComp Kids project team includes two Family Partners (EMC and SVR, both mothers of medically complex children) who have been involved with the study since its inception. These Family Partners are part of the research team and are remunerated for their time spent on the DigiComp Kids Project, according to recommendations set out by the Strategy for Patient-Oriented Research Networks [22]. The aim of including Family Partners within the co-design process as well as more broadly in the entire DigiComp Kids project is to ensure that our research prioritizes the concerns of medically complex children and their families, so that the DigiComp Kids intervention is relevant and useful to this community. Within the larger DigiComp Kids project, Family Partners have assisted in refining the research question, reviewing and contributing to a grant application to support this work, planning and preparing for the co-design day, and guiding the project development from their Steering Committee roles. In the overall project, Family Partnerships are situated at the ‘Collaborate’ and ‘Involve’ levels of the Levels of Patient and Researcher Engagement in Health Research, as identified by Manafo and colleagues [23]. Within the co-design portion of the project specifically, they have also taken on a dual role as consenting research participants by joining in and facilitating a portion of the co-design session, as well as critically revising both our co-design findings and this manuscript. During these activities, their level of engagement in the research process aligns with the ‘Participate’ stage [23]. Our team (including our Family Partners) chose to specifically separate the roles of our Family Partners into project team members (ongoing) and co-design participants (time-limited) by undertaking a process of informed consent for the co-design portion, in order for information shared through co-design to be protected by the rights afforded to research participants. Family Partners were under no obligation to participate in co-design and were informed that the choice not to participate would not affect their ongoing role as project team members, or their child’s medical care.

## Results

A Generative Co-Design Framework for Healthcare Innovation, presented here, was designed to be adaptable by healthcare innovators and end-users seeking to change a specific healthcare process or system. The importance of involving end-users in this process is critical— individuals living and working within a specific context have a deep understanding of the challenges they face and the intricacies of the environment in which these challenges are embedded. There exists a gap in the literature between the postulated benefits of health innovations, and the actual outcomes of these innovations when deployed in practice, which often fall short of their predicted benefits [24]. In particular, health system transformation via virtual care technology innovations, which is the focus of the DigiComp Kids project, is often undertaken by research teams without adequate attention paid to involvement of end-users in the design of innovations, resulting in a lack of adoption, scale, and spread [24, 25]. The complexity of both the health innovations themselves, as well as the environmental context in which they are implemented, results in interdependent human, socioeconomic, cultural, and technological factors that influence the outcomes of health innovation implementation [24].

These complexities have important implications for the implementation effectiveness of newly-developed health innovations, as relationships between humans and their contextual environment into which the innovation is introduced serve as mediating factors in how effective, acceptable, and usable those innovations are found to be [24, 26, 27]. Therefore, health innovations developed with closer attention to real-world concerns of end-users will be more likely to be usable and sustainable by clinicians, families, and patients, for improving or maintaining health [24].

**Fig. 1:**
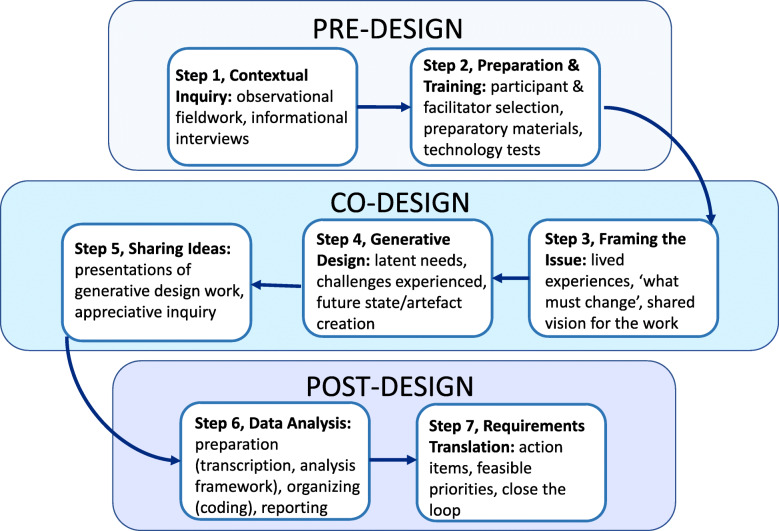
A Generative Co-Design Framework for Healthcare Innovation

A Generative Co-Design Framework for Healthcare Innovation is divided into seven steps within three stages— 1) Pre-Design, consisting of ‘Contextual Inquiry’ and ‘Preparation & Training’; 2) Co-Design, including ‘Framing the Issue’, ‘Generative Design’ and ‘Sharing Ideas’; and 3) Post-Design, consisting of ‘Data Analysis’ and ‘Requirements Translation’. Each stage is presented as a summary of activities and an example of how the stage was operationalized in the DigiComp Kids project. Figure 1 contains a summary of stages and their flow.

### Pre-design

#### Summary

The Pre-Design phase of the Generative Co-Design Framework for Healthcare Innovation includes the Contextual Inquiry (Step 1), and Preparation (Step 2). Contextual Inquiry aims to help the research team familiarize themselves with the current state, including the usual practices and processes of the healthcare setting in which co-design is to be implemented. Contextual inquiry may include employing ethnographic research methods such as observing the practice setting, including professionals within the setting and their workflows; conducting informational interviews to gain an understanding of end-user current challenges; or value stream mapping/workflow mapping with healthcare practitioners within the practice setting [28]. Shared team understanding of the current state is crucial for a productive co-design experience, and as such, even teams intimately familiar with the practice setting may wish to employ an abbreviated observational field experience, as appropriate. Next, Preparation aims to help future co-design participants and facilitators become acquainted with the project and begin to build rapport as a team. The Preparation phase may include the development and distribution of informational materials related to the project to future co-design participants and recruiting co-design session facilitators to help with co-design activities. Additionally, if co-design will be conducted virtually, testing the virtual system with future co-design participants will ensure connectivity and audiovisual issues are addressed before the co-design sessions are to take place, avoiding delays and frustrations during these sessions. Finally, regular group communication from the research team to co-design participants with informational materials and updates on preparations for co-design may help to build group engagement.

#### DigiComp Kids operationalization: pre-design

##### Step 1

During the Pre-Design phase of DigiComp Kids Co-Design, the lead author (MB) attended outpatient Complex Care Clinic appointments to obtain insights into the day-to-day rituals, habits, and workflows of clinic staff and patients. During these visits, field notes were kept detailing the needs of patients and families coming to clinic, clinic services provided, logistical, personnel, and space requirements for clinic functioning, and potential uses of virtual care technologies to facilitate clinic operations. Our research team (MB, MM, NC, AL) also conducted informal informational interviews with key stakeholders to discuss current state highlights, challenges, and visions for a technology-enabled future. In order to gain an understanding of diverse perspectives, we conducted these consultative meetings with a broad range of individuals, including parents of children with medical complexity, nurses, physicians, allied health professionals, as well as hospital and home-care administrators.

##### Step 2

In the Preparation phase, the DigiComp Kids research team met to collectively decide on which individuals should be invited to participate in co-design, with the aim of including participants who were representative of a wide variety of stakeholder groups. For the DigiComp Kids project, we selected 11 individuals to participate in our future co-design session who had experience in home and hospital-based care for medically complex children, system navigation, nursing practice support and leadership, as well as parents of medically complex children. After inviting participants to the project, we developed informational materials, including an agenda, a short pre-reading, and an instructional participant guide for the virtual platform to be used for co-design activities. These were distributed to future co-design participants to provide them with a background on the DigiComp Kids project, as well as the specific aims and structure of co-design. We also sent biweekly emails to future co-design participants with updates on project progress to keep them informed as co-design approached. Finally, each individual participant was contacted by the lead author (MB) ahead of the co-design day to gather informed consent for participation in co-design, answer outstanding questions, and conduct a technology test to ensure participants could log on and navigate the virtual platform to be used for co-design without issue.

A facilitation team was selected to assist with conducting synchronous activities during the co-design day, including five small group (CW, KL, CO, SM, CF) and three large group facilitators (MB, NC, MM). All facilitators were technologically savvy and had expertise in either relevant research methods or virtual healthcare design and implementation. Facilitators were briefed on the aims of the DigiComp Kids project and co-design day, and two mock co-design sessions were held with facilitators using the virtual co-design platform to practice the facilitation role before the co-design day.

### Co-design

#### Summary

Steps three, four, and five of the Generative Co-Design Framework for Healthcare Innovation (Fig. 1) comprise the co-design phase, wherein participants and facilitators engage in activities to conceptualize a future state of care. The steps within the co-design phase consist of: Framing the Issue (Step 3), Generative Design Work (Step 4), and Sharing Ideas (Step 5). In the DigiComp Kids project, these steps took place on a single day, however, the timeframe for other projects may vary, according to project needs. Due to the short time frame of a one-day, immersive, co-design event, the preparatory steps taken during pre-design featured prominently during the DigiComp Kids co-design phase. For example, the research team had already developed a deep understanding of the context in which we were working, due to the time spent observing Complex Care Clinic workflows and conducting informational interviews. This context was vital for helping participants to frame the issues discussed during co-design, and to engage deeply with them. Additionally, preparing co-design participants by briefing them on the aims of co-design, providing them with informational materials to become familiar with the DigiComp Kids project, and ensuring technology needed to access the virtual platform was working before attempting to login on Co-Design Day ensured a smooth co-design process.

Co-design work starts by framing the issue to develop a mutual understanding of lived experiences and challenges faced by participants in the current state, as well as a shared vision for the work. Participants willing to share stories of their experiences and obstacles faced in the current state are encouraged to do so, as these stories will help to ground the team in understanding what must change. The research team is encouraged to facilitate conversations with participants around a commitment to improvement (the goals of co-design), and a shared vision for the work (the plan to achieve those goals).

Next, participants and facilitators undertake creative generative design work. Generative techniques aim to both consider explicitly stated needs of participants, as well as to reveal latent needs — those that people are not yet aware of in their conscious minds, and therefore are not always readily expressed in words [29]. The rationale behind using generative techniques in relation to co-design is that if simply asked what is needed from a future healthcare system, participants may respond with solutions that improve current issues, but that do not respond to underlying root causes of problems. Root causes are not always readily identifiable— with generative techniques participants may be guided in stages to express deeper levels of knowledge about their experiences, challenges, and needs [29].

Many options exist for the selection of an appropriate generative technique, and the chosen exercise will depend on the needs of the research team and project. Examples of generative techniques used in co-design include a persona scenario exercise, which is undertaken to develop an understanding of participants’ experiences and challenges, as well as a vision for the future via the creation of an ideal state [13]; storytelling activities facilitated by illustrations and sketches [30]; or a creative prototyping exercise, in which participants create a physical manifestation of a concept or idea. The central concept to generative design is that participants have the opportunity to creatively draw upon their experiences, and using that experience, make something (an artefact) that illustrates a future state. In this way, designers can harness the expertise of participants to both learn about the past, as well as to shape the future.

The artefacts created—be they stories, physical prototypes, illustrations, or other creative outputs—are then shared amongst the larger team in the final step of the Co-Design phase. The creation and sharing of artefacts allows participants to access their experiences in new and creative ways, and reflect on why they chose to create what they did [29]. Within the Sharing Ideas sessions and associated dialogue, the research team should pay attention to similarities and differences of artefacts created by different groups, points of emphasis by participants, and stated priorities for the future state of healthcare. In order to capture the breadth of knowledge shared, it is recommended that these Sharing Ideas sessions are audio-recorded, with participant consent.

#### DigiComp Kids operationalization: co-design

##### Step 3

In the co-design phase of the DigiComp Kids project, our Family Partners and two expert clinicians from the Complex Care Team presented accounts of challenges they had encountered in the current state. These stories were shared with the intention of building empathy, understanding the need for clinical change, and cultivating a shared sense of purpose among group members. Next, members of Ontario Health (OTN) presented case scenarios of healthcare solutions that they had previously helped to develop, in order to give examples of success stories and speak to the scope of change required for program implementation. During these case scenarios, technology was emphasized as an *enabler* of care, but participants were cautioned that implementing a new technology solution would not be sufficient to transform care in most cases, without consideration of context, workflows, and system integration.

##### Step 4

Subsequently, participants split into small groups, each led by a facilitator, to begin generative design work. For our generative design activity, we selected a persona scenario exercise, where participants worked together to develop a fictitious character that was representative of others ‘like them’. To facilitate this, we grouped participants with similar experiences together (e.g. hospital-based healthcare practitioners), in order to encourage the development of detailed and authentic personas.

A ‘persona’ is a detailed and realistic character that is representative of participants’ stakeholder group [13]. Personas are meant to be fictional, yet draw on the expertise of the people creating them in order to construct a character that is representative of a ‘typical’ end-user for that group [31]. Small group facilitators guided the development of personas using a worksheet (Additional file 3). Guiding questions asked included highlights and challenges of persona’s roles, their comfort levels and experiences with tablets, vital signs devices, and other technology types, and important tasks that they perform in their work with medically complex children.

During scenario work, groups selected an important challenge that their persona encountered, and then imagined a ‘future state’ where care would be delivered differently, to solve that challenge. To distil details of persona-technology interaction within the scenario, as well as requirements for a future state, guiding questions were used to direct group discussion. Specific questions asked by facilitators included “If your persona had remote access to healthcare providers and services, what would be different about the way that care is provided? How would this help to solve the challenge you’ve selected? What technologies are needed to support this change? How would this change the way that information is provided, care is coordinated, families are supported?”. The scenarios constructed by the participant pairs allowed for exploration of how personas might interact with features of a future health system. This exploration of human-system interaction is termed “contextmapping” [29] and is a vital component of designing a healthcare innovation that is suitable for the environment into which it will be implemented [24]. Within DigiComp Kids co-design, facilitators guided participants to define what would be different in the future state, which formed the basis of considerations for innovation design.

##### Step 5

Finally, participants re-convened in a large group for the Sharing Ideas sessions, where they each presented their persona scenario exercise in turn and spoke to the group about their experiences with the exercise. Facilitators and other group members used a process of appreciative inquiry to highlight the positive aspects of the persona scenario exercises, and to expand on and help to develop these ideas. Questions asked by facilitators and other group members during the audio-taped Sharing Ideas sessions helped presenting participants to highlight points of emphasis and importance, as well as areas of uncertainty encountered during the persona scenario exercise.

### Post-design

#### Summary

The Post-Design phase of the Generative Co-Design Framework is comprised of Step 6, Data Analysis, and Step 7, Requirements Translation. During the Data Analysis phase, the research team sorts and transcribes data, organizes data by distilling themes, and engages in a process of checking in with co-design participants to ensure that the distilled themes match with participant views of relevant and important topics. The aim of data analysis is to capture the most pertinent and significant ideas, which will then be used to form the basis of the healthcare innovation.

In the Requirements Translation phase, the research team uses the themes derived from co-design to decide on priorities for the innovation, plans the innovation based on what can reasonably be achieved, and finally closes the loop with co-design participants and stakeholders to identify plans for moving forward with the innovation. To accomplish this, the team starts by assigning action items to each theme and sub-theme from the co-design findings (i.e. actions that would be required to actualize the theme). For example, if co-design participants emphasized the need for an accessible source of personal health information, including current lab results and care plans, a secure patient portal, compatible with mobile devices, may be designed. Next, the research team reviews necessary action items to decide on innovation priorities by determining: which items already exist (and can be leveraged), which items are infeasible to develop, and which items should move forward to form the basis of the innovation [13]. The final step in co-design is to circle back to co-design participants and stakeholders invested in co-design to inform them of the results of the design process, and the plan of action moving forward.

#### DigiComp Kids operationalization: post-design

##### Step 6

Our team selected directed qualitative content analysis as our data analysis technique, and moved through three phases of preparation, organizing, and reporting results [32–34]. The lead author (MB) collated and transcribed materials from large group co-design presentations, persona scenario audiotaped presentations and small group worksheets, as well as personal memos and reflections from the co-design process. Next, transcripts were read several times to facilitate a clear understanding of the data and emerging themes, and particular attention was paid to articulations of ‘what must change’ by participants. The lead author (MB) then developed an initial coding framework, based on the research question, which was to investigate the optimal processes, features, and workflows for a virtual care intervention [24]. Definitions were developed for each of these categories, and the first ten pages of the transcript were coded independently by the lead author (MB) and a senior member of the research team with qualitative expertise (NC). These authors then met to compare and refine initial codes, after which time the lead author (MB) continued to code the rest of the transcript. Finally, codes were summarized under the categories of processes, features, and workflows, and themes and sub-themes were distilled from the data. These themes and sub-themes were collated into a summary document and shared with DigiComp Kids co-design participants through a process of member-checking. Participants were asked to reflect on the summarized content of co-design as to whether it ‘fit’ with their interpretation of the day, and to share edits, questions, or memos that came to mind as they read through the summary documents. These additions and edits were incorporated into the final summary co-design findings document.

##### Step 7

During DigiComp Kids Requirements Translation, the research team met to discuss the co-design findings and steps needed to realize each of the themes. Next, the research team communicated with leaders in the hospital, home care, and technology development sectors to identify if there existed tools or technologies that could be leveraged to meet any of the requirements for DigiComp Kids. With this new knowledge, the research team met several more times to design the processes, workflows, and features for DigiComp Kids, based on the requirements articulated by co-design participants and within the constraints of what was possible for the timeline, budget, and scope of work for the project. Co-design participants and healthcare leaders were once again thanked for their contributions to the design of this project, and the final project design was communicated in a news brief.

## Discussion

Healthcare innovation is essential for finding strategies to balance costs and quality of care in a climate of healthcare resource restriction juxtaposed against ongoing efforts to improve excellence in care delivery and quality of life for patients. While evidence of successful healthcare innovation programs exist, the majority of newly developed healthcare innovations are not routinely integrated into care. The term ‘pilot-itis’ was coined to represent the plethora of innovation attempts that begin and end with a pilot or beta model [35]. To combat the pilot-itis that plagues the healthcare innovation sphere, incorporation of co-design methods may assist healthcare innovators, researchers, clinicians, and quality improvement specialists in developing useful, manageable, and sustainable healthcare innovations. The purpose of this work was to develop and apply A Generative Co-Design Framework for Healthcare Innovation in our project, DigiComp Kids.

Family Partners, who have been involved with the DigiComp Kids study since inception, were critical team members in ensuring the success of our co-design project. In being fully immersed in DigiComp Kids for over 1 year’s time, both Family Partners used their expertise during co-design to support the overall goals of the DigiComp Kids project. For example, since the DigiComp Kids study seeks to design a virtual care program, one concern that our team had was that co-design participants would focus solely on the types of technology needed to implement this care model, as opposed to focusing on the necessary workflows, processes, and system requirements needed to support a technology-enabled care model. Knowing this, during our co-design day, our Family Partners were able to clearly articulate where they believed technology would help in caring for medically complex children at home, in addition to where other low-technology or technology-free options would be just as useful, and what would be needed to support these options. Because of this, the entire co-design team was able to focus on supporting system-level change as the focus of co-design, with technology acting as an enabler of care.

Based on our co-design experience, we encourage healthcare innovators and research teams to involve patient partners in projects from the earliest possible date to ensure immersion in the project, shared understanding between researchers and patient partners about the goals of the study, and full participation. In the DigiComp Kids study, our Family Partners worked with the research team to design the flow and activities of the co-design phase and reported satisfaction with the process. Additionally, although our Family Partners already encouraged other co-design team members to think of both technology-focused and low-technology solutions for care without our direction to do so, we could have asked that this be an explicit role of Family Partners during co-design. Healthcare Innovation teams may want to consider pre-assigning roles to individuals for co-design activities, for situations such as this, as we feel this would strengthen the implementation of the framework.

### Strengths and limitations of our framework

Situated in the conceptual areas of patient engagement frameworks and health innovation design, A Generative Co-Design Framework for Healthcare Innovation offers a method for research teams focused on engagement of end-users in creative innovation design work. One of the strengths of this framework is the emphasis on engagement of end-users, including patient partners and other stakeholders, as a vital component of designing relevant, acceptable, useable health innovations. Use of co-design strategies for healthcare innovation includes the lived experiences of end-users in research, generates ideas for patient-focused service improvements, empowers the included groups, and tailors interventions to end-user requirements thus increasing the likelihood of their adoption and integration [36–39]. Successful examples of co-design have been demonstrated in existing literature [14, 15], wherein strategies such as patient journey-mapping, experience-based surveys, and workshops have been utilized to improve end-user adoption and integration of program services.

Another highlight of this framework is the emphasis on the incorporation of creative strategies for idea generation, while allowing for flexibility for research teams to customize these creative strategies to their needs. The ability to generate many alternative solutions to a problem, to approach problems with an open mind, and to tolerate ambiguity and persist in seeking novel solutions with merit are all attributes of individuals’ creative personalities that strongly affect the likelihood of successful innovation [40]. Thus, the central place of creative strategies within A Generative Co-Design Framework for Healthcare Innovation is a key strength in its construction as a tool for health innovation.

Additionally, there are some limitations of the framework that must be considered. The first is that our framework relies on the in-depth participation of key end-users to shape the future state of care, which may result in an over-reliance on the perspectives of a dedicated group of few end-users, who may not be representative of the larger population of interest. In combating this limitation, healthcare innovation teams may want to consider selecting a diverse array of end-users to participate, including those working in diverse roles, from differing age, cultural, and gender identities, and with varying years of professional experience, in order to make the application of the framework more generalizable. While highly contextualized and intimate personal knowledge that comes from individual end-users is key to the creation of a useable healthcare innovation, these needs must be balanced against the creation of an innovation that will be applicable beyond a small group.

A second potential limitation of our Framework is that some end-users may have concerns about speaking up around personal challenges encountered in the current system. End-users are often highly entrenched in the system which they are being asked to critique during co-design, creating the potential for them to approach co-design in an overly cautious manner. In healthcare settings, fear of retaliation is a well-known barrier to speaking up with critiques of the healthcare system or context in which individuals work [41, 42]. To confront this potential limitation, healthcare innovation teams are encouraged to invest time in establishing trusting relationships with participants and maintaining an environment of openness and acceptance during co-design. Some potential strategies to accomplish this are informal individual meetings with participants before co-design, as well as team building exercises such as those described under the ‘Framing the Issue’ step.

Finally, we acknowledge that the co-design process described within our framework is time- and labour-intensive. In the context of healthcare innovation, we appreciate that many innovation projects are undertaken by clinicians, quality improvement specialists, and researchers who are already pressed for time and resources. However, we have demonstrated that the actual co-design engagement from participants can be successfully undertaken in a single day, with the proper preparations being taken during the Pre-Design phase. This is in contrast to other methodologies such as Experience-Based Co-Design, in which engagement sessions are typically run over multiple days. Therefore, for teams in which engaging co-design participants over multiple sessions may be difficult, our framework may offer an advantageous alternative to others.

## Conclusion

Co-design of healthcare innovations represents an opportunity to leverage the knowledge, experiences, and insights of end-users to achieve impactful innovations in healthcare contexts. For healthcare innovators seeking to expand their innovations beyond the pilot phase, A Generative Co-Design Framework for Healthcare Innovation provides guidance on incorporating end-user voices in innovation design. This Framework contributes to the literature in the patient engagement field by offering a new category of patient engagement frameworks focused on engaging end-users in the creative process of health innovation. Healthcare innovators, applied health science researchers, clinicians, and quality improvement specialists may wish to refer to the Framework and worked example presented here in order to elicit the viewpoints of end-users while distilling practical considerations for healthcare innovation and design.

## Supplementary Information

**Additional file 1: Table 1.** Definitions of Terminology Used.

**Additional file 2.** GRIPP2 Long Form.

**Additional file 3.** Persona Development Worksheet.

## Data Availability

The datasets used and/or analysed during the current study are available from the corresponding author on reasonable request.

## Abbreviation

OTN: Ontario Telemedicine Network
