# Supplementary material for: A generative co-design framework for healthcare innovation: development and application of an end-user engagement framework
Source: Res Involv Engagem. 2021 Mar 1;7:12. doi: 10.1186/s40900-021-00252-7 (PMC7923456; doi:10.1186/s40900-021-00252-7)
Supplement: Supplementary file 1 — Additional file 1: Table 1. Definitions of Terminology Used. [file 40900_2021_252_MOESM1_ESM.docx]

**Additional File 1**

**Table 1: Definitions of Terminology Used**

| **Term** | **Definition** |
| --- | --- |
| Healthcare innovation | Aims to develop new healthcare service and delivery methods, products, policies, technologies, or systems, with a view toward improving people’s health (1). |
| End-user | Individuals who have a vested interest in the outcome of innovation design, development, and implementation. |
| Family members | Parents or caregivers responsible for caring for children with medical complexities; may or may not be biologically related. |
| Processes | The way that care is integrated and organized as a system, including the coordination of care between sectors. |
| Features | The components that make up the hardware and software of the intervention, as well as capabilities of the intervention for delivering virtual care. |
| Workflows | The way that individuals or families use the system to provide care, including the roles and responsibilities of the people involved. |

1. World Health Organization. Promoting Health Through the Lifecourse. In: Group WHI, editor.
