# Supplementary material for: A generative co-design framework for healthcare innovation: development and application of an end-user engagement framework
Source: Res Involv Engagem. 2021 Mar 1;7:12. doi: 10.1186/s40900-021-00252-7 (PMC7923456; doi:10.1186/s40900-021-00252-7)
Supplement: Supplementary file 3 — Additional file 3. Persona Development Worksheet. [file 40900_2021_252_MOESM3_ESM.docx]

**Additional File 3**

**Persona Development Worksheet**

| **My name is:**  ______________________ | | **I am _____ years old**  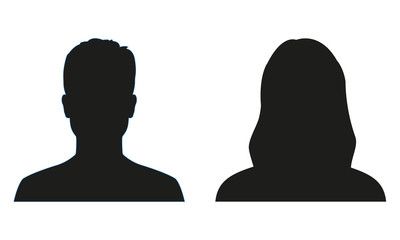  **I am a**: Man Woman | |
| --- | --- | --- | --- |
| **My caregiver role:** | | | |
| **Any other jobs that I have:** | | **Other important caregivers/team members:** | |
| **I typically provide ___ hours of care to a child with medical complexities in a**  Day Week | | | |
| **Important care tasks that I do are…** | | | |
| **Typical assistive technologies that I interact with/use to provide care:** | | | |
| Wheelchair |  | Non-invasive ventilation |  |
| Home oxygen |  | Invasive ventilation |  |
| Tracheostomy |  | Long-term IV/port |  |
| G/J tube feeds |  | CSF shunt |  |
| Hearing aids |  | Other: |  |
| Dialysis |  | Other: |  |
| **Highlights of my role…** | | | |
| **Challenges/frustrations of my role…** | | | |
| **My comfort level with technology:**  Not comfortable  A little comfortable  Mostly comfortable  Very comfortable | | | |
| **The technology devices I use most often are:** | | | |
| Mobile phone |  | Tablet |  |
| Land line |  | Remote monitor (e.g. Fitbit or vital signs monitor) |  |
| Desktop computer |  | Webcam |  |
| Laptop computer |  | Other: |  |
| **I prefer to communicate with other caregivers/team members:**  In person  On the phone  Over email  By video call | | | |
| **Other important details about me**: | | | |
